# The RELATE model: strategies to effectively engage healthcare organisations to create amenable contexts for implementation

**DOI:** 10.1108/JHOM-08-2020-0335

**Published:** 2021-06-24

**Authors:** Aoife De Brún, Eilish McAuliffe

**Affiliations:** UCD Centre for Interdisciplinary Research, Education and Innovation in Health Systems (UCD IRIS), School of Nursing, Midwifery and Health Systems, University College Dublin , Dublin, Ireland; School of Nursing, Midwifery and Health Systems, University College Dublin , Dublin, Ireland

**Keywords:** Change management, Implementation, Influence, Research work

## Abstract

**Purpose:**

The field of implementation science has emerged as a response to the challenges experienced in translating evidence-based practice and research findings to healthcare settings. Whilst the field has grown considerably in recent years, comparatively, there is a conspicuous lack of attention paid to the work of *pre-implementation* , that is, how we effectively engage with organisations to support the translation of research into practice. Securing the engagement and commitment of healthcare organisations and staff is key in quality improvement and organisational research. In this paper the authors draw attention to the pre-implementation phase, that is, the development of an amenable context to support implementation research.

**Design/methodology/approach:**

Drawing from examples across an interdisciplinary group of health systems researchers working across a range of healthcare organisations, the authors present a reflective narrative viewpoint. They identify the principal challenges experienced during the course of their work, describe strategies deployed to effectively mitigate these challenges and offer a series of recommendations to researchers based on their collective experiences of engaging in collaborations with healthcare organisations for research and implementation. This reflective piece will contribute to the narrative evidence base by documenting the challenges, experiences and learning emerging from the authors’ work as university researchers seeking to engage and collaborate with healthcare organisations.

**Findings:**

The RELATE model is presented to guide researchers through six key steps and sample strategies in working to secure organisational buy-in and creating a context amenable to implementation and research. The six stages of the RELATE model are: (1) *R* ecognising and navigating the organisation's complexity; (2) *E* nhancing understanding of organisational priorities and aligning intervention; (3) *L* everaging common values and communicating to key individuals the value of implementation research; (4) *A* ligning and positioning intervention to illustrate synergies with other initiatives; (5) *B* uilding and maintaining credibility and *trust* in the research team; and (6) *E* volving the intervention through listening and learning.

**Research limitations/implications:**

The authors hope this guidance will stimulate thinking and planning and indeed that it will encourage other research teams to reflect and share their experiences and strategies for successful engagement of organisations, thus developing a knowledge base to strengthen implementation efforts and increase efficacy in this important enterprise.

**Originality/value:**

Researchers must relate to the world’s everyday reality of the healthcare managers and administrators and enable them to relate to the potential of the research world in enhancing practice if we are to succeed in bringing the evidence to practice in a timely and efficient manner. Climates receptive to implementation must be developed incrementally over time and require actors to navigate messy and potentially unfamiliar organisational contexts. In this paper, the often invisible and lamentably underreported work of how we begin to work with healthcare organisations has been addressed. The authors hope this guidance will stimulate thinking and planning and indeed that it will encourage other research teams to reflect and share their experiences and strategies for successful engagement of organisations, thus developing a knowledge base to strengthen implementation efforts and increase efficacy in this important enterprise.

## Introduction

The field of implementation science has emerged as a response to the challenges experienced in translating research findings and evidence-based practices to healthcare settings. Implementation research is defined as “ *the scientific study of methods to promote the systematic uptake of research findings and other evidence-based practices into routine practice* ” ( Eccles and Mittman, 2006 ). Whilst this has been an important field in ensuring implementation of evidence-based practices and is supported through methods to help foster and effectively leverage conditions for change, comparatively there is a conspicuous lack of attention paid to the work of *pre-implementation* , that is, how we effectively engage healthcare organisations as external partners in research collaborations that involve the implementation of an intervention and support the translation of research into practice. Many implementation strategies, such as those featured in the Expert Recommendations for Implementing Change ( Powell *et al.* , 2015 ), are narrowly focused on strategies related to a specific intervention or innovation rather than on the broader initial goal of engaging an organisation and securing organisational agreement and commitment to research collaboration. This is a critical but largely ignored part of the research and implementation process. Securing the engagement and commitment (i.e. “buy-in”) of healthcare organisations and staff is key in quality improvement and organisational research, particularly where organisations are committing to a change in practice or service delivery.

In this paper we contend that an intervention is more than the intervention implementation itself but must be considered as dependent on an *amenable context* . We consider an amenable context as one where there is broad organisational support for the collaboration in advance of specific interventions being prepared or deployed. It differs from readiness for change as it constitutes the step prior to the initial engagement of the staff who will be implementing the change and is focused on exploring the possibility of collaboration. An amenable context is cultivated through the development of effective, mutually beneficial inter-organisational collaborations, often between a university research group and a health organisation or system, which require considerable time, effort, and skill to initiate and maintain. The aim of this article is to draw attention to this pre-implementation phase, that is, the development of an amenable context. Drawing from examples across an interdisciplinary group of health systems researchers (with expertise drawn from the disciplines of business, health economics, health informatics, psychology, sociology, law, medicine, public health, nursing and geography) working across a range of healthcare organisations from hospital networks to large acute hospitals, to regional, district and local healthcare organisations, as well as with non-governmental organisations, charities and community groups, in both high-income and low and middle income settings, we identify the common principal challenges experienced during the course of our work. This work includes engaging organisations in the co-design, deployment and testing of a range of interventions aimed at enhancing care quality, safety, equity, integration of care, service (re)configuration, and leadership and teamwork. In this paper, we describe strategies deployed to effectively mitigate these challenges and offer a series of recommendations to researchers based on our experiences (drawn from routine project meetings, supervision and mentoring meetings with PhD and postdoctoral research fellows, and interdisciplinary research seminars and problem-solving workshops of engaging in collaborations with healthcare organisations for research and implementation). This reflective piece will contribute to the narrative evidence base by documenting the challenges, experiences and learning emerging from our work as university researchers seeking to engage and collaborate with healthcare organisations.

### Challenges in engaging healthcare organisations

Healthcare organisations are complex, multifaceted structures, with larger organisations often characterised by multiple layers of clinical and corporate management. Different health systems will be organised in different ways, and this understanding of the system will influence the level at which it may be appropriate to seek support and engagement. Regardless of system design, depending on the level of the system at which one is aiming to seek engagement, the context (both wider macro context and local meso and micro contexts) must be considered. For instance, when seeking engagement of a healthcare organisation, such organisations often form a component of a larger regional or national system and thus are influenced by these broader external factors (as well as internal factors). An individual organisation may be further sub-divided by speciality or profession, adding challenges when planning initial engagement strategies with colleagues in healthcare settings. It is not sufficient to focus only on the target of the intervention and the immediate culture or context within which this target is situated. Following the successful engagement of an organisation, successful implementation of any intervention will require preparatory work with stakeholders operating in the broader context of the organisation (e.g. a hospital network or trust).

In advance of being granted permissions to access the target audience for the intervention, it is necessary to first ensure that the decision makers in the system and/or organisation understand the intervention, appreciate the unique value it will contribute, trust the architects and implementers (often researchers external to the organisation), and believe that the intervention has a reasonable chance of success. These are all necessary pre-cursors to the support or buy-in that is often cited as being essential to the success of healthcare interventions.

Diverse working cultures can exist between and within healthcare organisations. The micropolitics that characterise these structures mean that the “unwritten rules”, that is the cultural norms, interests, values and power relationships, can be difficult to determine from the outside ( Langley and Denis, 2011 ). Failing to effectively determine and negotiate these ways of working and engage in an appropriate and timely manner with individuals can risk alienating staff if they feel ignored or circumvented ( Smollan, 2011 ; Nilsen *et al.* , 2019 ). This is a particular hazard given the top-down hierarchy pervasive in healthcare. Power and authority in the healthcare hierarchy is well-documented within and between professional groups ( Crowe *et al.* , 2017 ; Blanchfield and Biordi, 1996 ), and clinician–manager relationships can also be fraught, with tensions and dissatisfaction in relationships widely reported to the extent that frontline staff can feel “rigorously divided from management” ( Davies *et al.* , 2003 ; Edwards *et al.* , 2003 ; Ennis and Harrington, 1999 ).

Healthcare organisations are complex and dynamic entities, comprising individual agents acting and reacting in ways that are not predictable. On-going self-organising, adaptive processes necessitate that priorities and ways of working are in flux, and thus, individuals respond based on their experiences and perceptions. Effectively navigating organisational politics and understanding who the key influencers are is crucial to ensuring engagement and buy-in. Inadvertently aligning with the “wrong” camp, for example, managers but not senior clinicians, or senior clinicians whilst ignoring managers, could be detrimental to implementation success. Thus, spending time understanding the needs, values, perspectives and motivations of key local stakeholders will be crucial to understand how to successfully collaborate to ensure engagement and commitment of influencers who can help drive the implementation.

Whilst identifying and accessing relevant stakeholders may be one challenge, given the pressures and demands on healthcare staff, it is not unusual to encounter resistance to engagement and change. Self-interest, stress, uncertainty, personality and different perspectives on issues can all contribute to resistance to change and implementation of new practices ( Curtis and White, 2002 ). Additionally, a new research collaboration or implementation of a new intervention may be perceived as competing with, or detracting from, others in the system. This can also promote resistance, scepticism and cause “intervention fatigue”.

### Strategies employed to engage organisations and stakeholders

Given the multiple factors requiring consideration, we have summarised some of these issues and suggest six key steps and sample strategies to attempt to mitigate and address challenges to promote the successful (and on-going) engagement of healthcare organisations in research and implementation partnerships ( Table 1 ). Column 3 of Table 1 provides some practical examples that have had positive results in our work across several organisations. A detailed explanation and contextualised discussion of these findings is presented below. Steps 1–4 focus on recommendations to *effectively engage* healthcare organisations in research collaboration and to support the translation of research into practice, while steps 5 and 6 are focused on strategies to *ensure the continued engagement* of the organisation through the maintenance of an effective collaborative relationship.

#### Recognising and navigating the organisation's complexity

McFadden *et al.* (2006) describe the importance of an enthusiastic leader who buys into implementation of new initiatives as key to achieving higher levels of collaborative implementation. This individual can exert influence in terms of identifying and helping to overcome internal barriers. Whilst the organisational chart will be informative and crucial in obtaining essential approvals, the individuals who play boundary spanning roles across departments or professional groups may not necessarily be represented on such charts, but can have an influential impact on promoting communication and making links across the organisation ( Farmer, 2008 ). An organisation's face value (as represented by the organisational chart) rarely gives a true picture of how that organisation functions or the myriad roles individuals, who are key to the organisation's functioning, may be performing. Where no pre-existing relationships exist with organisation members, early engagement with key stakeholders is vital to generate buy-in and commitment. Garvelink *et al.* (2015) found that “pre-engagement” with healthcare providers in advance of research (by informing clinical directors of the trial and asking them to provide letters of intent to support the research) affected subsequent participation in the research where there were no pre-existing ties between the research team and organisations. This exposure to researchers and timely flagging and initiation of relationships supported and enabled subsequent engagement. Although such engagement takes time and resources and ideally needs to occur before any funding has been secured for the research, health research funders are becoming more attuned to the value of this engagement, with many now seeking evidence of this in applications for funding. Preparing a clear, concise project brief and requesting time to present the intervention at management meetings and holding open drop-in sessions to communicate information to staff were effective strategies we have employed in our work to generate interest and understand how best to navigate the organisation to obtain the requisite engagement. In one large multisite study, we requested time to present at meetings of the senior executive team of the hospital management group. This allowed us to present the potential value of the work, gauge interest from key leaders and engage with them directly, addressing queries and concerns. Whilst this is a crucial initial step, we also encourage researchers to maintain regular contact and updates with those at the highest levels of management to ensure the initiative remains on the agenda and to support subsequent steps of the process (e.g. building and maintaining trust in the research team).

#### Enhancing understanding of organisational priorities and aligning intervention

In healthcare organisations, systems are complex and dynamic, and a main challenge is understanding and reconciling diverse, sometimes conflicting, priorities and goals. A beneficial mechanism both to promote consensus and to instil ownership in solutions, is the use of collaborative and co-design approaches, whereby healthcare staff can work with researchers to co-develop and craft a shared vision and set of priorities for a planned research collaboration. The relationship between clinicians and managers is central to the success of improvement and change efforts ( Davies *et al.* , 2003 ), and thus, this co-design can be very effective in fostering a shared mental model across disparate staff groups and the research team ( Pallesen *et al.* , 2020 ). It has the added benefit of developing a shared understanding about objectives and outputs, and promotes clarity around the value of outcomes to various stakeholders. Where co-design may not be feasible, demonstrating “fit”, that is, how the planned collaboration can support an organisation or team in achieving their goals or vision, will be important to explicitly illustrate the shared benefits of a new partnership. In previous work, we have spent time learning about the organisation from individuals operating at various levels of the system and from the strategy and policy documents of the organisation and the wider health system. This has enhanced our ability to identify and effectively target communications to critical influencers and decision makers.

#### Leveraging common values and communicating to key stakeholders the value of implementation research

Accessing key stakeholders within organisations and communicating the value of implementation research to these individuals of various roles and levels in the organisation is only possible with the insight of who these people are, their values and their objectives. Learning from discussions in Step 1 is key to informing who should be contacted in Step 3. Organisational structures and hierarchies set the scene for discussion and negotiation ( Caffrey *et al.* , 2016 ). Leveraging previous or existing working relationships or organisational institutional agreements (e.g. a hospital group or healthcare trust agreement with a university) or establishing a new memorandum of understanding can present valuable insight into the operations of an organisation. As previous work has described, failure to consult those who expect to be consulted about a planned change or collaboration can destabilise a collaboration effort from the outset, even causing staff to become “incensed when they were not consulted about a change or some aspect of it” ( Smollan, 2011 , p. 842). Fitzgerald *et al.* (2002) describe various types of opinion leaders that can effectively support implementation: those who share information across networks, those considered to have expertise and local credibility and those with strategic management and micropolitical skills. Critical to implementation success is finding common ground with such opinion leaders and ensuring they become advocates for the intervention. Accessing key people in the organisation and communicating the potential value of the collaboration to various groups and levels in the organisation through tailored messaging is key. However, various groups may be more or less receptive to different messages and messengers, and thus, attention to the type of stakeholders (e.g. opinion leaders) accessed and consulted is crucial to ensure widespread support and create a more amenable context for successful collaboration and implementation. Some of our most successful collaborations have involved the team inviting key stakeholders to become more directly involved as an investigator, member of the research team, project team or advisory/expert group member to explicitly demonstrate the team's commitment and openness to true collaboration.

As we outlined earlier, health systems are inherently complex, and organisations are often nested within a larger regional or even national network. Engaging with stakeholders at this level is also key. Garnering support from patient organisations and collaborating as partners with patients and patient representatives or groups further strengthens the credibility and perceived potential impact of the collaboration.

#### Aligning and positioning intervention to illustrate synergies with other initiatives

In healthcare settings, the quality improvement and staff development space are already crowded with various, often competing, interventions. Rather than competing, it is important to demonstrate how the new intervention can complement, support and/or advance an existing effort in the system. Establishing complementarities and synergies and explicitly aligning the intervention to the organisation's broader objectives and strategic plan will help to positively frame the intervention, making its relevance and potential impact clear. Alignment differs from appropriateness as the latter is defined as the perceived fit, relevance, or compatibility of the intervention for a given setting or perceived fit to address a particular issue ( Proctor *et al.* , 2011 ) but ensuring alignment refers to the positioning of the collaboration and/or intervention related to other organisational initiatives, policies or strategies. The deliberate identification of synergies and opportunities will illustrate the alignment of the collaboration to organisation goals and objectives. Alignment and appropriateness are related concepts, and we contend that ensuring alignment will enhance perceptions of appropriateness. In our previous work, planning collaborations for the future introduction of a team-training intervention, we emphasised how this would be timely in aligning to a new national policy in recognising the need for multidisciplinary team training to promote integrated care. Furthermore, we created alignment at the organisational level by highlighting how the team training intervention could support the organisation's in-house quality improvement training by creating a platform for teams to work together to improve quality.

#### Building and maintaining credibility and trust in the research team

Building credibility and trust in the research team is especially important where there is no track record of collaboration or contact. Behavioural integrity is fundamental, that is, ensuring alignment between words and deeds (what is promised and what is delivered) ( Simons, 2002 ; Simons *et al.* , 2012 ). Trust is a key antecedent of commitment to a collaborative working relationship. Mayer *et al.* (1995 , p. 721) define trust as the “willingness of a party to be vulnerable to the actions of another party based on the expectation that the other will perform a particular action important to the trustor, irrespective of the ability to monitor or control that party”. Regarding research that (at a minimum) involves staff involvement and where there is at least a possible impact on patients, this mutual trust is central to promoting engagement for collaboration. Researchers can further support the development of trust by adding value to organisations through engaging in smaller projects that benefit the partner organisation. We have worked to develop trust through developing evidence summaries valuable to the organisation's planning and decision-making, analysing and feeding back data, assisting in writing up local projects for publication, and supporting healthcare staff in developing funding proposals and business cases. Committing resources to the research partnership, for instance, embedding researchers in the organisation to enable data collection and support real-time data analysis and feedback and thus creating formal boundary spanning roles can be powerful enablers of collaboration ( Rycroft-Malone *et al.* , 2015 ; Long *et al.* , 2013 ). This indicator of commitment through dual roles creates opportunities to disseminate information and prepare appropriate implementation strategies through a more nuanced understanding of the organisational and research context ( Rogers *et al.* , 2020 ). However, the availability of resources (or lack thereof) may constrain feasibility of this approach when operating across multiple organisational contexts.

#### Evolving the intervention through listening and learning

Staff engagement is promoted when individuals feel listened to and can have a meaningful influence on a change process. However, resistance to change and to engagement is common in healthcare due to the busy nature of the work and the multiple demands on staff. Resistance is multifaceted and multidimensional and most frequently considered as resistance to management, but actors at all organisational levels can demonstrate resistance to change ( Smollan, 2011 ). A key factor in addressing resistance to change is “understanding the attraction for change rather than battling resistance” ( Plsek and Wilson, 2001 ). Understanding the tension for change can be a more powerful influencing force in drawing people to a change and can also support more effective message tailoring to key individuals and groups. Resistance is a valuable source of feedback and provides an opportunity to harness the negative energy to evolve an intervention, adapting it to address concerns in a positive and constructive manner that will improve the prospects of implementation success. In our previous work, we have held open discussions with stakeholders (including those resistant and those eager to implement) to understand the issues and challenges and seek solutions. Asking open questions and hearing from resistors on what they feel needs to be changed, adapted or what additional resources may be required can help to surface and identify barriers. The opportunity to voice concerns is valued by staff and often those in the room are best placed to identify and co-create solutions.

## Conclusion

It is widely recognised that translational gaps might be narrowed by bringing the users and producers of research together through collaborative, mutually beneficial relationships ( Rycroft-Malone *et al.* , 2015 ). Such relationships are critical not only to enabling the conduct of effective, high quality and meaningful research, but to translating the findings of such research into practice. This pre-implementation work or relationship building is necessarily a dynamic and adaptive process occurring in highly complex and dynamic contexts. Climates receptive to implementation must be developed incrementally over time and require actors to navigate messy and potentially unfamiliar organisational contexts. Just as architects need to understand builders and vice versa, researchers must relate to the everyday reality of the healthcare professionals and enable them to relate to the potential of research in their practice if we are to succeed in bringing the evidence to practice in a timely and efficient manner. In this paper, we have addressed the often invisible and lamentably underreported work of how we begin to work with healthcare organisations. The complexity of healthcare organisations demands that that we “grapple with the world we actually inhabit, not the one we wish we did” ( Braithwaite *et al.* , 2018 ). This forces us to attend to the many barriers and challenges inherent when we endeavour to partner with healthcare organisations to conduct research on implementation and change.

Jagosh *et al.* (2012) found that partnership synergy is both an outcome and a context for partnership development. This suggests that when collaborations generate positive outcomes (e.g. implementation success for improved outcomes), those outcomes generate new synergy and can foster momentum for further change. Thus, alignment and development of shared mental models are crucial for partnership working and collaboration. Collaboration and building relationships for effective implementation are most appropriately considered a skill that requires effort and practice. When change is planned or occurs, this inevitably is seen as threatening to some, serving to challenge existing beliefs and behaviours ( Asnawi *et al.* , 2014 ). Navigating this context successfully requires emotional intelligence and self-awareness, as well as political skills. These frequently labelled “soft skills” are underappreciated and undervalued in this type of work. In reality, there is “nothing soft about the soft skills” ( Goldman and Wong, 2020 ), and instead they can constitute “the hard stuff” ( Covey, 2003 ) and are core competencies when working to engage people and organisations. These skills are usually developed and honed over time through extensive experience.

Whilst we have delineated the significant challenges and some strategies that benefitted our work, such work can never be considered concluded. Our argument is that these efforts allowed us to form the bedrock for an on-going collaboration where engagement strategies became focused on implementation and more targeted intervention efforts. Finally, whilst we have offered guidance for researchers, we do not contend that this will be sufficient to successfully engage all organisations. As we are warned, learning cannot be transformed into a narrow recipe associated with a target ( Plsek and Wilson, 2001 ). Indeed, the on-going COVID-19 pandemic demonstrates the need to be flexible and responsive to the wider context, adapting as appropriate. We believe these steps can be adapted to support remote engagement with healthcare organisations, although clearly, the demands on healthcare currently require careful consideration of the potential benefits of research in the context of staff time and resources. We hope this guidance will stimulate thinking and planning, and indeed, that it will encourage other research teams to reflect and share their experiences and strategies for the successful engagement of organisations, thus developing a knowledge base to strengthen implementation efforts and increase efficacy in this important enterprise.

## Figures and Tables

**Table 1:** Strategies to address challenges in engaging healthcare organisations (the *RELATE* model)

Challenges	Strategies to address challenges	Practical examples
1. *Recognising* and navigating an organisation's complexity and hierarchy to identify influencers	Ensuring familiarity with formal organisational chart Leveraging working relationships to create opportunities for informal conversations with staff to understand key people in the network and understand norms	Requesting time to briefly introduce intervention at existing decision-making fora, e.g. management team, clinical leaders, nursing management, health and social care professionals meetings Holding open lunchtime sessions to introduce the intervention to staff Meeting with existing contacts in the organisation to identify key influencers/opinion leaders and spending time explaining the rationale and evidence for the intervention (recognising that they will influence others to participate)
2. *Enhancing* understanding of the priorities of different stakeholder groups and demonstrating how the intervention helps address those priorities	Understanding and documenting diverse priorities Seeking alignment to organisation's vision and strategy Engaging stakeholders in co-design to ensure intervention and approach to implementation is fit-for-purpose Bestowing ownership through involvement	Rather than presenting a finished product, bringing evidence and ideas to a forum that engages the intended targets of the intervention to co-design or adapt both the content and the approach to delivery. This co-design process surfaces the diverse priorities in the organisation, provides the research team with an enhanced understanding of the organisational dynamics and the potential barriers or sticking points and how to overcome these Leveraging institutional agreements/memoranda of understanding to demonstrate contribution to a larger vision
3. *Leveraging* common values and communicating to key individuals the value of implementation research	Mapping key stakeholders to promote/authorise planning collaboration and engagement Recognising and addressing conflicting values (real and perceived) in the organisation Tailoring messages to each group based on diverse interests: identifying unique value to individuals/teams and shared value to wider teams/organisation Ensuring continued relevance through regular updates to the management team	Nuancing presentations on the intervention to demonstrate the research team's understanding of value system and challenges of the particular group/audience, e.g. a management team may be more focused on the end result of improved patient safety whereas a clinical team, whilst striving to achieve patient safety may be more concerned with the processes, roles and responsibilities in the team
4. *Aligning* and positioning intervention to illustrate synergies with other initiatives	Understanding and mapping other improvement initiatives in the system. Identifying and emphasising complementarities, synergies and alignment between efforts rather than competition Ensuring explicit alignment to the organisation's objectives/strategic plan	Engaging in sense-making. Resisting the temptation to compete with other interventions, instead identifying synergies and complementarities and demonstrating how the intervention can add value to on-going work/other initiatives in the organisation
5. Building and maintaining credibility and *trust* in the research team	Demonstrating an understanding of the value system through work in the preceding phases Acknowledging the challenges and constraints that exist within the organisation Adding value to the organisation through feeding back evidence, findings and results (or other agreement mechanisms of communication) Ensuring a certain level of authority/credibility required among team members meeting with hospital management Considering resource commitments Ensuring behavioural integrity	Investing the time and resources to build a relationship for the longer term, not just for the project Increasing understanding of research and the potential of the research team to help the organisation attain its objectives Helping with other organisational initiatives, e.g. help staff to publish previous work, develop a research framework for a project, write a proposal for funding, etc. Providing timely and practical feedback as the research progresses – demonstrate value early to help bring more people on board ( Powell *et al.* , 2015 ) Recognising that seniority confers credibility, ensuring that senior researchers in the team are visible and engaged in supporting more junior researchers Behaving with integrity, maintaining a neutral stance, refraining from engaging in organisational politics, etc.
6. *Evolving* the intervention through listening and learning	Recognising that some staff will feel threatened by change in the status quo. (listening to and learning from resistance) Listening to concerns, demonstrating how intervention addresses these and being willing to adapt intervention to better address needs	Utilising resistance as a positive force to adapt an intervention and increase its fit-for-purpose Harnessing evidence-based implementation strategies to support adaptation (e.g. Powell *et al.* , 2015 ) Demonstrating that the team understands and takes seriously the concerns of individuals and groups also helps build the credibility of the research team
